# Is there any clinical relevant difference between non mosaic Klinefelter Syndrome patients with or without Androgen Receptor variations?

**DOI:** 10.1038/s41598-017-03371-y

**Published:** 2017-06-13

**Authors:** Umberto Valente, Cinzia Vinanzi, Savina Dipresa, Riccardo Selice, Massimo Menegazzo, Massimo Iafrate, Carlo Foresta, Andrea Garolla

**Affiliations:** Unit of Andrology and Reproductive Medicine, Department of Medicine, University of Padova, 35122 Padova, PD Italy

## Abstract

Klinefelter Syndrome (KS) is the most common chromosomal disorder in men leading to non-obstructive azoospermia. Spermatozoa can be found by TESE in about 50% of adults with KS despite severe testicular degeneration. We evaluated AR variations and polymorphism length in 135 non-mosaic KS patients, aimed to find possible correlation with clinical features, sex hormones and sperm retrieval. Among 135 KS patients we found AR variations in eight subjects (5.9%). All variations but one caused a single amino acid substitution. Four variations P392S, Q58L, L548F, A475V found in six patients had been previously described to be associated with different degrees of androgen insensitivity. Moreover we observed in two patients Y359F and D732D novel variations representing respectively a missense variation and a synonymous variation not leading to amino acid substitution. All the Klinefelter patients with AR gene variations were azoospermic. Spermatozoa were retrieved with TESE for two men (40%), sperm retrieval was unsuccessful in other 3 patients. This is the only study reporting AR variations in KS patients. Relevant clinical differences not emerged between AR mutated and not AR mutated KS patients, but does each variation play an important role in the trasmission to the offspring obtained by ART in this patients?

## Introduction

Klinefelter syndrome (KS) is the most frequent sex chromosome disorder, occurring in about 1 boy in 600 and diagnosed in 11% of azoospermic men^1^. Patients with Klinefelter syndrome (47,XXY) are characterized by eunuchoid body proportions, gynecomastia, small firm testes and azoospermia.

Testicular biopsies from Klinefelter patients show mixed areas with Sertoli-cell-only tubules and sclerotic or hyalinized tubules as well as interstitial Leydig cell hyperplasia. However, scattered areas with focal spermatogenesis can be seen even in non-mosaic patients^2^ (3% of patients is mosaic 46,XY/47,XXY). Testicular sperm recovery is estimated in 50% of adult KS men by means of TESE and used as the sperm source for intracytoplasmic sperm injection (ICSI)^3^.

KS patients can have normal total testosterone values, but there are many KS presenting hypogonadism and poor virilization. Androgens play an important role in the prenatal and pubertal virilization of the external genitalia in 46,XY fetuses. The effect of androgens is mediated by the androgen receptor that plays a critical role in male sexual differentiation, development and maintenance of secondary male characteristics and the induction and maintenance of spermatogenesis^4^. AR, as a ligand-activated transcription factor, is activated by binding either of the androgenic hormones testosterone or dihydrotestosterone^5^. In humans, AR is a 110 kD protein composed of 919 aminoacids that is encoded by the AR gene located on the X chromosome at Xq11-12^6, 7^. AR gene polymorphisms and mutations cause a wide spectrum of phenotypic abnormalities in 46, XY subjects. Androgen insensitivity syndrome (AIS) comprises two clinical subgroups: complete AIS (CAIS) and partial AIS (PAIS)^8^. Missense mutations lead to partial androgen insensitivity syndrome (PAIS), characterized by the presence of abnormal genital development in a 46,XY individual with normal testis development and partial responsiveness to age-appropriate levels of androgens. There are no available data on the prevalence of PAIS in the general population. CAIS is a disorder of sex development (DSD) characterized by the presence of female external genitalia, ambiguous genitalia or variable defects in virilization in a 46,XY individual with absent or partial responsiveness to age-appropriate levels of androgens. The CAIS phenotype is associated with an AR mutation that completely disrupts AR function; target cells do not respond to testosterone or dihydrostosterone (DHT). The estimated incidence is between 1/20,000 and 1/99,000 live male births. In the AR gene, four different types of mutations have been detected to generate defective AR: (I) single point mutations resulting in aminoacid substitutions or premature stop codons; (II) nucleotide insertions or deletions most often leading to a frame shift and premature rumination; (III) complete or partial gene deletions; and (IV) intronic mutations causing alternative splicing^9^. Defective AR may include loss or gain of function AR alterations leading different implication in spermatogenic function. The human AR gene contains two polymorphic (CAG)n (polyGln/polyQ) and (GGC)n (polyGly/polyG) repeat sequences in exon 1. The number of polyQ and polyG repeats is 21.6 ± 3.3 (range, 9–31) and 17.4 ± 1.4 (range, 8–21) respectively, as reported in fertile controls in a previous work of our group^10^.

PolyQ tract length has been reported to affect AR activity. Shorter polyQ tract can enhance the critical intramolecular N-C terminal interaction of AR, allowing the response to lower androgen concentrations associated with higher levels of specific p160 coactivators^11^.

The framework already complex of AR gene is made even more complicated in KS patients, given that: (1) there are two X chromosomes, (2) the X chromosomes may be the same or different depending on the type inheritance, (3) inactivation of the X chromosome.

In the present study we evaluated AR mutations and CAG/GGC polymorphism in KS patients in order to find possible correlations with clinical features, sex hormones and sperm retrieval.

## Results

We retrospectively evaluated the presence of AR mutations in 135 non-mosaic KS patients referred at the Unit of Andrology and Reproduction Medicine of Padova for fertility problems. Six AR variations were found in eight infertile men affected by non mosaic Klinefelter Syndrome. AR variation frequency was of 5.9% (8/135).

Table 1 reports clinical, hormonal and anthropometric parameters of the eight AR mutated KS patients (mean age 35.3 ± 10.51) and the 127 Klinefelter (mean age 31.5 ± 8.5) without AR variations. Comparison between Klinefelter patients with and without AR gene variations was than performed for the above mentioned parameters. Anthropometric characteristics were similar in AR mutated and non mutated KS patients. Sex hormones were comparable in the two groups, while PTH levels resulted significantly higher in non mutated KS patients (p = 0.0047), despite the main value was in the normal range. Serum 25-OH Vitamin D, calcium and phosphorus did not differ between two groups and resulted in the normal range. Also metabolic parameters were in the normal range and not different between two groups. No difference was observed for left and right testicular volumes between AR mutated and non mutated KS patients.

**Table 1:** Clinical characteristics observed in 8 AR mutated and in 127 non mutated KS patients.

Variable	Pt 1	Pt 2	Pt 3	Pt 4	Pt 5	Pt 6	Pt 7	Pt 8	Mean ± SD	KS with no AR mutations (127) Mean ± SD
Age (year)	18	43	41	39	34	23	50	35	35.3 ± 10.51	31.5 ± 8.5
Height (cm)	177	175	189	175	191	180	185	170	180.2 ± 7.4	181.3 ± 7.9
Weight (kg)	66	78	88	103	127	75	87	50	84.2 ± 23.3	85.4 ± 16.8
BMI (kg/m²)	21	25.4	24.6	33.6	34.7	23.1	25	17	25.5 ± 5.9	26.0 ± 4.6
Waist (cm)	84	92	96	110	124	85	101	71	95.3 ± 16.5	98.9 ± 14.9
Arm Span (cm)	180	176	190	178	194	181	186	174	182.37 ± 7.01	181.7 ± 14.3
Testicular volume L (cc)	1.7	1.5	2.8	1.5	7	1.7	1	1.1	2.28 ± 0.6	2.0 ± 0.7
Testicular volume R (cc)	1.9	2.3	3.8	2	3	1.8	1.5	1.1	2.5 ± 0.7	2.1 ± 0.8
Total Cholesterol (mg/dl) (r.v 120–200)	154	211	178	191	231	174	200	157	190.5 ± 32.2	194.8 ± 44.2
HDL (mg/dl) (r.v > 40)	62	53	59	57	32	90	51	63	59 ± 24.2	50.0 ± 15.3
Triglycerides (mg/dl) (r.v < 150)	46	112	66	101	235	40	73	38	96.5 ± 93.7	119.6 ± 163.6
Glycemia (mg/dl) (r.v 50–110)	77	92	85	80	74	88	76	77	78.7 ± 6.2	79.4 ± 13.1
Insulin (mU/l) (v.r < 29.1)	17,7	11,2	11,4	10.8	35,8	4,9	2	2	11.1 ± 16.4	9.8 ± 7.8
Total Testosterone (nmol/l) (r.v 10–29)	8.9	10.5	16.7	12.8	9.2	12.1	13.5	13.2	11.4 ± 5.17	10.5 ± 4.9
LH (IU/l) (r.v 1–9)	31.5	19.5	19.6	20.6	17.6	25.4	17.5	28.7	21.8 ± 9.4	19.6 ± 6.8
FSH (IU/l) (r.v 1–8)	85.4	33.7	34.7	35.8	19.1	39.3	23.5	62.5	36.1 ± 19.6	33.6 ± 12.9
Estradiol (pmol/l) (r.v 25–130)	51	119	156	98	94	84	103	115	99 ± 13.1	106.1 ± 34.2
PSA (g/l) (r.v < 4)	0.59	0.49	4.44	0.51	0.29	0.25	1.16	0.84	0.63 ± 0.44	0.69 ± 0.99
PTH (ng/l) (r.v. 17–73)	21.4	41.4	29	34.3	22	25.5	23	73	35.8 ± 24.7*	69.3 ± 43.2
25-hydroxyvitamin D3 (nmol/l) (r.v. 50–125)	69	68	45	49.8	30	62	58	28	51.4 ± 17.2	52.3 ± 27.3
Calcium (nmol/l) (r.v. 2.10–2.55)	2.54	2.45	2.38	2.41	2.44	2.39	2.53	2.49	2.4 ± 0.1	2.4 ± 0.1
Phosphorus (nmol/l) (r.v. 0.87–1.45)	0.84	0.8	0.8	0.81	0.8	1.05	0.65	1.11	0.91 ± 0.2	1.0 ± 0.2

**Figure 1:**
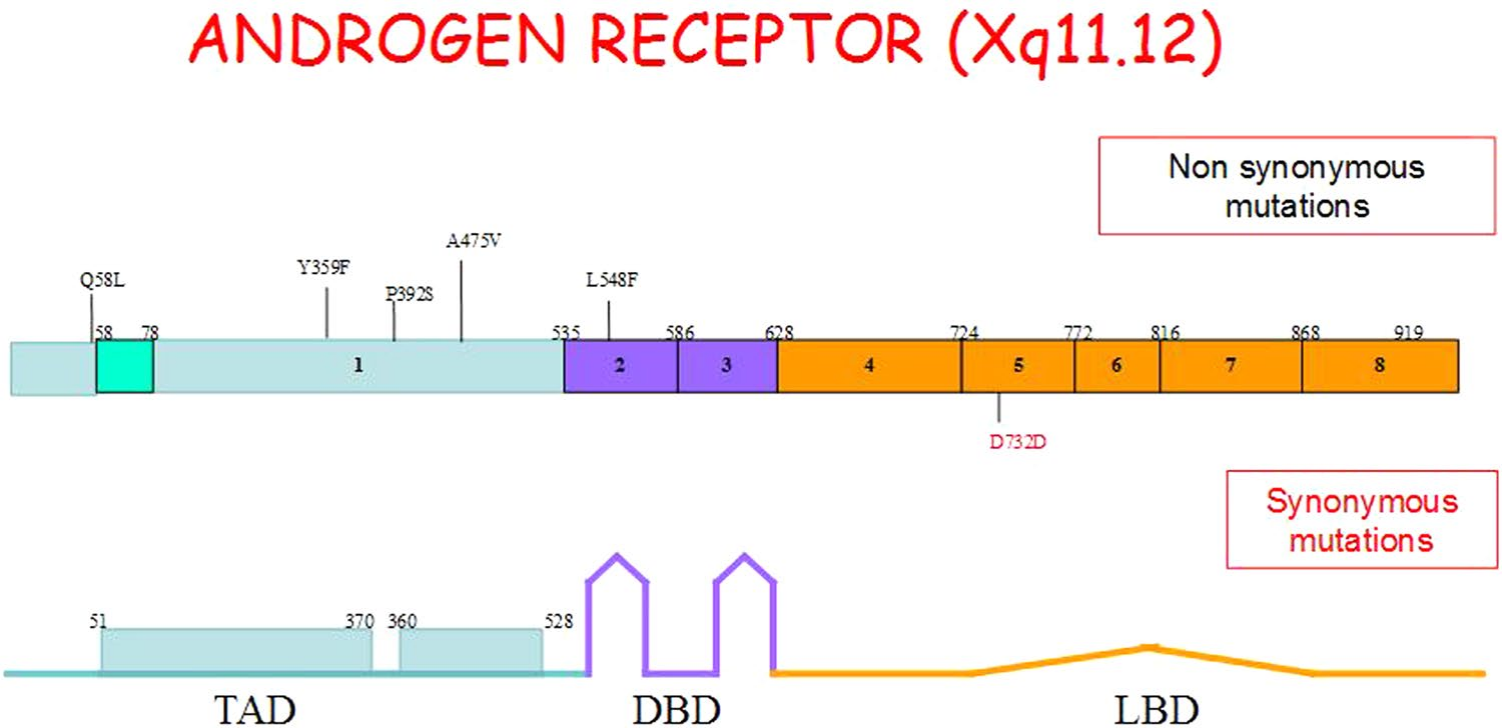
In the upper part of the figure are show the 8 exons of the Androgen Receptor gene with the position of each gene mutation found in our study (missense mutations in black, synonymous mutation in red). The lower part of the figure indicating the correspondence between exon and AR protein domains: exon1 coding for TAD (transactivation domain), exons 2 and 3 coding for DBD (DNA binding domain), exon 4–8 coding for LBD (ligand binding domain).

Table 2 shows androgen receptor variation type observed in each Klinefelter subject. In the same table are reported the RefSNP (rs numbering) of each variation according to NCBI dbSNP, the corresponding amino acid substitution and the number of CAG and GGC repeats. Finally we summarized heterozygous or homozygous status of the mutation for each patient and the previously phenotype reported by literature. All variations but one caused a single amino acid substitution. Four variations P392S, Q58L, L548F, A475V (six patients) had been previously described (www.androgendb.mcgill.com) and shown to be associated with different degrees of androgen insensitivity. Two patients (patient 2 and 4) carrying the same AR variation P392S, other two patients (patient 3 and 5) carrying the same AR variation Q58L. Variation Y359F (found in patient 1) and variation D732D (found in patient 8) represent novel finding of missense and synonymous variation (not leading to amino acid substitution) respectively. Five patients (patient 2, 3, 4, 5, 6) had heterozygous variations, while three showed homozygosity for the variation (patient 1, 7 and 8). Of the six variations, four (66%) were localized in the TAD protein domain, one (16%) in the DBD protein domain and one (16%) in the LBD protein domain (LBD) (Fig. 1). CAG and GGC repeats in these patients were in the range of normality.

**Table 2:** AR variation found in our patients: type of mutation, exon and domain involved, acronym that identifies the variation (rS), number of CAG and GGC repetition, number of allele mutated, corresponding phenotype when available.

Patients	AR mutation	Exon	Protein domain	Rs Dd SNP	CAG	GGC	State of mutation	Type of mutation	Clinical features reported in literature
1	Y359F	1	TAD-Nter	753297907	19	18	homozygous	missense	Not studied
2	P392S	1	TAD-Nter	201934623	24/26	17/18	heterozygous	missense	MAIS*, PAIS°, Testicular cancer
3	Q58L	1	TAD-Nter	200185441	24/26	17/18	heterozygous	missense	MAIS*
4	P392S	1	TAD-Nter	201934623	22/23	17/18	heterozygous	missense	MAIS*, PAIS°, Testicular cancer
5	Q58L	1	TAD-Nter	200185441	22/23	17/18	heterozygous	missense	MAIS*
6	L548F	2	DBD	139524018	17/18	17/18	heterozygous	missense	MAIS*, PAIS°
7	A475V	1	TAD-Nter	200390780	21	17/18	homozygous	missense	MAIS*
8	D732D	5	LBD	199940567	19	18	homozygous	synonymous	Not studied

All Klinefelter patients included in the study had normal analysis of Y chromosome microdeletions. We found in ten of 127 non mutated KS patients rare sperm after centrifugation (7.8%). All the Klinefelter patients with AR gene variation were azoospermic and no immature germ cells were found in semen, even after centrifugation. Among these patients, five undergone testicular biopsy and the results of sperm retrieval obtained by bilateral surgery are reported in table 3. This procedure allowed us to retrieved and cryopreserved sperm in two of five patients (40%). Patient 1 (variation AR Y359F and CAG 19 GGC 18) had bilateral sperm retrieval despite a low testicular volume (left and right testicular volumes 1.7 and 1.9 cc respectively). Patient 5 (AR variation Q58L and CAG 22/23 GGC 17/18), had a testicular volume higher than expected in KS subjects (volume 7cc) and a right testicular volume of 3cc. In this patient we retrieved sperm from the left testis.

**Table 3:** Sperm retrieval in 8 KS mutation and type of mutations.

Patients	Testicular Biopsy	Sperm retrieval (SR)	SR Unilateral/Bilateral	AR Mutation
1	Yes	Yes	Bilateral	Y359F
2	No			P392S
3	Yes	No		Q58L
4	No			P392S
5	Yes	Yes	Unilateral (Left)	Q58L
6	Yes	No		L548F
7	No			A475V
8	Yes	No		D732D

## Discussion

47 XXY karyotype is the most common chromosomal disorder in men and accounts for 11% of all men with azoospermia. More than 95% of individuals are unable to have biological children by natural means^12^. Despite non-mosaic Klinefelter patients show the same karyotype, they have variable phenotypic characteristics^13^. The reason for this heterogeneous clinical finding is unclear, but it has been suggested that it may be caused by various genetic and epigenetic effects^14, 15^. Moreover, this heterogeneous clinical variability could may be caused by low androgen levels or to a reduced androgen sensitivity. On the same basis, a lower fertility potential has been supposed in those patients with more phenotypic severity, however this theory has not been confirmed yet. Despite low testosterone levels are associated with reduced fertility in 46, XY males, there is no similar evidence in KS patients.

Mutations in the AR gene have been reported in infertile males^16–18^ in association with a wide array of phenotypic abnormalities^13, 19^, but only few data are available for Klinefelter patients. To our knowledge, only one study has investigated the presence of AR mutations in a little group of Klinefelter subjects, showing no mutations in this cohort^20^. It is possible, however, that mutations in these patients remained undetected because the low sensitivity of the SSCP method (Single Strand Conformation Polymorphism) used for the analysis or because the little number of included patients.

In our study, 135 Klinefelter subjects were evaluated by Sanger sequencing for variations in the AR gene and we found nucleotide variations in 8 of them (5.9%). Of the six variations identified, four (66%) were localized in the TAD domain, one (16%) in the DBD domain and one (16%) in the LBD domain. Mutations in the LBD or DBD domains have been reported in more severe forms of androgen insensitivity syndrome while variations in the TAD domain appear to be often associated with mildest phenotypes, such as isolated male infertility^21^. Four variations P392S, Q58L, L548F, A475V had been previously described^22–27^, while the detection of Y359F and D732D variations represents a novel finding.

Beside testicular failure and sex hormones abnormality, Klinefelter syndrome is frequently associated with alterations of lipid, glucose and bone metabolism. In our study the analysis of these parameters showed no difference in testicular volumes, sex hormones and anthropometric parameters of KS patients with and without AR variations. Also metabolic features were similar in two groups, except for PTH levels. Despite in the normal range, circulating PTH resulted significantly higher in patients without AR variations compared with mutated KS subjects. No difference was observed regarding Vitamin D, calcium and phosphorus levels. PTH significantly lower in the group of KS patients with AR mutation may simply be an expression of an important numerical difference between the two groups.

Despite our findings suggest that AR seems not to affect spermatogenesis, sex hormones and metabolic pattern in KS, this is the first report showing AR variations in these patients. On the other hand, we cannot exclude that AR variations in Klinefelter Syndrome may have an impact on the fertility because of the following reasons: (i) different types of mutation (synonymous variation generally does not damage the protein; missense mutation may have a loss or a gain of the receptor function; frameshift or stop codon mutation, usually disrupt the receptor structure and function) usually leads to different phenotypes; (ii) X inactivation status could influence phenotypic expression of the AR mutated X. As KS patients have two X chromosomes, the mutational inactivation of AR gene on the mutated allele may be compensated by the presence of the wild type AR gene on the transcriptional active X allele; (iii) homozygous or heterozygous status of the mutation (in homozygous patients the mutated allele is expressed despite the X chromosome inactivation) may have different impact on the testicular function^28–30^.

To further point out the complexity of this issue, we underline that two patients with the same heterozygous variation (Q58L) showed a different outcome at testicular biopsy. Patient 5 had successful sperm retrieval while patient 3 had not. We can speculate that this discrepancy depends on a different inactivation pattern of the extra X allele. It is possible to suppose that in the patient 5 the X-inactivation acts by silencing the X-allele carrying the mutation, while in the patient 3 the silencing affects the wild type allele. In addition, patient 1, carrying in homozygous state the Y359F variation (not previously described in literature) and showing particularly small testicular volumes (each testicle was <2cc), had bilateral sperm retrieval. In this patient, since the effect of the variation was not masked by the X-inactivation of the extra X allele (homozygous), it is possible to suppose that this variation may have not an impact or even a protective effect on spermatogenesis.

In conclusion, AR variations can be identified in a significant percentage of KS patients. From this study we cannot drive final conclusions regarding the role of AR variations in KS subjects, however it is possible that these alterations may have a significant impact on the clinical feature and spermatogenic impairment of patients. Therefore, we hope that further studies involving larger cohort of subjects will be performed to clarify these aspects to evaluate the presence of AR gene variations in KS patients and to study the relevance of X-inactivation in mutated subjects aimed to extend the knowledge on this topic. Finally, the knowledge of AR variations could be useful to better determine the reproductive risks during the genetic counselling of those KS patients undergoing assisted reproduction. Their female offspring carriyng AR variation maight transmit the mutated allele to the 50% of her offspring, resulting in a failure of normal masculinization of the external genitalia in chromosomally male individuals (Morrys Syndrome). This failure of virilization can be either complete or partial depending on the amount of residual androgen receptor function, the partial androgen insensitivity syndrome ranging from mildly virilized female external genitalia to mildly undervirilized male external genitalia.

## Methods

### Ethical approval

The study has been approved by the Ethics Committee of the University Hospital of Padova (Protocol number 2357P) and each participant gave his written informed consent. The study has been conducted in accordance with the principles expressed in the Declaration of Helsinki.

### Subjects

The patient provided written consent to diagnosis and anonymous divulging of medical data for scientific and research purposes, according to the local ethics committee. Patients with more than one supernumerary X chromosome, mosaicisms, or with endocrine dysfunction different from hypogonadism, and subjects assuming testosterone replacement therapy or any other drug were excluded from study. Subjects evaluation included complete medical history (pubertal history, lifestyle, physical activity, smoking, alcohol misuse), physical examination (weight, height, body mass index, waist circumference, arm span, measurement of testicular volume using Doppler ultrasound scrotal), lipid and glucose metabolism (total cholesterol, HDL, triglycerides, fasting glucose), complete blood count (CBC), serum insulin, PSA, hormone levels (LH, FSH, Total Testosterone, Estradiol, Prolactin), phosphocalcic metabolism (PTH, 25-OH-Vitamina D, serum calcium and phosphorus), and analysis of microdeletions of the Y chromosome. Each subject performed two seminal examination and, in case of azoospermia, a testicular biopsy. Finally, we compared KS subjects with and without AR mutations.

### Metabolic parameters

Plasma glucose was determined by enzymatic test (COBAS C720, Roche Diagnostic, Indianapolis, Indiana, USA); glycated hemoglobin was determined with HPLC procedure (Adams HA-8180 Arkray, Kyoto, Japan); total cholesterol was measured by enzymatic colorimetric test (CHOD-PAP, COBAS C720, Roche Diagnostic, Indianapolis, Indiana, USA); HDL cholesterol, and triglyceride concentrations were measured by enzymatic colorimetric test (COBAS C720, Roche Diagnostic, Indianapolis, Indiana, USA). Creatinine was determined by the Jaffe’ method. Calcium and phosphate were analyzed by enzymatic colorimetric test (BAPTA, COBAS C720, Roche Diagnostic, Indianapolis, Indiana, USA). PSA was determined by chemiluminescent immunoassay (Architect Immunoassay analyzer, Abbott laboratories, Abbott Park, Illinois, USA).

For all the parameters, the intra- and inter-assay coefficients of variation (CV) were\8 and 10%, respectively.

### Hormone assays

Serum concentrations of total T and E2 were determined by competitive chemiluminescent enzyme immunoassay, using commercial kits (Immulite 2000, Siemens Healthcare Diagnostics Products). Serum concentrations of SHBG, FSH, and LH were determined by chemiluminescent enzyme immunometric assays (Immulite 2000). Detection limits and intra-assay and interassay coefficients of variation were 15 ng/dL (0.5 nmol/L), 7.2%, and 8.2% for T; 15 pg/mL (55 pmol/L), 9.9%, and 16% for E2; 0.02 nmol/L, 2.7%, and 5.2% for SHBG; 0.1 IU/L, 3.2%, and 4.1% for FSH; and 0.05 IU/L, 3.4%, and 7% for LH.

### Ultrasonography (US)

CDUS scrotal evaluation was performed by two expert sonologists using an AplioTM XV echo color Doppler device (Toshiba, Tokyo, Japan) with a high resolution multifrequency linear probe (6–13 MHz). The testicular volume was estimated using the ellipsoid formula (length × width × height × 0.52), and the varicocele was assessed in standing position after the Valsalva’s maneuver in accordance with Sarteschi’s classification^31^.

### Genetic analysis

Mutation analysis of AR gene was performed on genomic DNA, extracted from peripheral blood leucocytes using a commercial DNA isolation kit according to the manufacturer’s instructions (Qiamp DNA Blood Mini Kit; Qiagen).

DNA was amplified by polymerase chain reaction with previously published primers covering exons 1–8^32^. Polymerase chain reactions were performed under standard conditions with the addition of 8% dimethyl sulfoxide in the reaction. Exon 1 was amplified to generate partially overlapping fragments while exons 2–8 were amplified using intronic primers. All PCR fragments were then analyzed by directly sequencing on ABI Prism sequencer (Applied Biosystems). Sequence analysis was performed using the gap4 software of the Staden package (http://staden.sourceforge.net/). Protein sequence comparison was performed using CLUSTALW computational analysis (www.ebi.ac.uk/clustalw/) (data not shown). Prediction of the possible impact of amino acid substitution on the structure and function of the protein was performed using the online free software Polyphen-2 (http://genetics.bwh.harvard.edu/pph)^33^. Aminoacid numbering is based on NCBI reference sequence NM_000044.2.

### Statistical analysis

Data in tables are presented as mean ± standard deviation (SD) of the mean. Descriptive statistics were used to summarize all demographic and clinical characteristics of the patients.

An appropriate statistical test was used to assess the relationship of various clinical characteristics with outcome. A paired ‘t’-test was used to compare means of all quantitative variables measured between the two studied groups. P-value smaller than 0.05 was considered as statistically significant.
